# Sm/Co Magnetic Materials: A Recycling Strategy Using Modifiable Hydrophobic Deep Eutectic Solvents Based on Trioctylphosphine Oxide

**DOI:** 10.3390/ijms241814032

**Published:** 2023-09-13

**Authors:** Nikita A. Milevskii, Inna V. Zinov’eva, Arina V. Kozhevnikova, Yulia A. Zakhodyaeva, Andrey A. Voshkin

**Affiliations:** Kurnakov Institute of General and Inorganic Chemistry, Russian Academy of Sciences, Leninsky Prospekt 31, 119991 Moscow, Russia; mna@igic.ras.ru (N.A.M.); iz@igic.ras.ru (I.V.Z.); ak@igic.ras.ru (A.V.K.); voshkin@igic.ras.ru (A.A.V.)

**Keywords:** solvent extraction, deep eutectic solvent, trioctylphosphine oxide, SmCo magnets, metal separation

## Abstract

Hydrophobic deep eutectic solvents (HDES) are widely used as extractants. Usually, when preparing HDES, only the extraction ability of one component is taken into account, with the second serving as an “inert” component, whose effect on the extraction process is not taken into account. The present study demonstrates the possibility of controlling the selectivity of a hydrophobic deep eutectic solvent based on trioctylphosphine oxide (TOPO) by varying the substance that acts as a hydrogen bond donor, but which does not have an extractive ability. In the course of the work, the influence of the “inert” component on the physicochemical and extraction properties of HDES was confirmed by experimental, spectroscopic, and also calculation methods. A number of phenols with different structural features were chosen as the HDES’ hydrogen bond donors to modify: phenol (Ph), para-tert-butylphenol (PTBP) and thymol (Th). Using the example of separation of the Sm/Co pair, the influence of the structure of a hydrogen bond donor on the extraction ability of a hydrophobic deep eutectic solvent was established, where the degree of extraction of Sm (III) increased in the series Th:TOPO < PTBP:TOPO < Ph:TOPO. HDES based on TOPO and phenols can potentially be used to separate Sm and Co from the process leach solutions generated during the hydrometallurgical processing of waste SmCo magnets.

## 1. Introduction

With the development of industry, samarium–cobalt magnets have become more sought after, due to their excellent magnetic properties and their high temperature and corrosion resistance [1,2]; however, the increasing amount of spent magnetic materials and waste from magnet production is prompting researchers to actively develop recycling technologies to recover, purify and further use metals such as Sm and Co [3].

The continuous development of recycling technologies creates a great demand for the development of effective methods of separating complex metal mixtures. In addition, the current trends in industry are to reduce the toxicity of waste and improve the safety of production to reduce the ecological burden on the environment. The most popular method of metal ion separation is liquid–liquid extraction, which has advantages such as a simple hardware design and relatively low energy costs. Many approaches have been proposed to replace traditional organic extraction systems with more environmentally friendly ones, to reduce the toxicity and increase the purity of the obtained substances. In particular, two-phase aqueous systems have great possibilities for extraction [4,5], but they have not been widely used because of the difficulties associated with back-extraction.

A major milestone was the development of ionic liquids (IL), which are widely used as highly selective extractants [6]. There are, however, a number of disadvantages of IL, such as their high viscosity [7] and often toxicity [8], which make it difficult to use them widely in industry. Hydrophobic deep eutectic solvents represent, from a practical point of view, an alternative to IL. Like widely used hydrophilic DES [9,10,11], they are less toxic, are easily prepared from available components and often do not require dilution for use. HDES consist of a hydrogen bond donor (HBD) and an acceptor (HBA), which are linked by hydrogen bonding and van der Waals interactions. These intermolecular interactions contribute to lowering the crystallization temperature of the mixture, so that the HDES are characterized by a liquid aggregate state. The last few years have seen a growth in the number of studies on the preparation and use of HDES as extractants for metal ions, and the main direction of this field has already been established [12,13,14,15].

One of the most popular trends has been the preparation of HDES based on components with known extraction properties paired with various available HBD or HBA that practically do not affect the extraction ability. The number of works using quaternary ammonium bases as HBAs and nonionic compounds as HBDs is striking. Such HDES have excellent physico-chemical properties and a selectivity towards a number of platinum and transition metals [16,17,18]. HDES based on quaternary ammonium compounds belong to Type III according to the classification proposed in the article [19]. More often, however, in liquid–liquid extraction, preference is given to neutral extractants, since they are easier to regenerate and exhibit better physico-chemical properties. Later proposed by Abranches et al. [20], Type V HDES consist only of neutral components and have also been actively studied recently for the above reason. Trioctylphosphine oxide (TOPO) is actively used as HBA in Type V HDES’ preparation because of its known selectivity towards rare earth metals (REM) [19]. Usually, a cheap and accessible substance with low toxicity is chosen as the second component. Various mixtures of TOPO with menthol [20], dodecanol [21] and other substrates have also been investigated [15,22].

However, there are several papers in the scientific literature that describe any effect of the component on HDES’ properties. A number of works are devoted to the study of the influence of the nature of donors on solid–liquid equilibrium (SLE) phase diagrams. For example, Martins et al. [23] have widely studied the effect of fatty acid homology on SLE phase diagrams with thymol and menthol. The authors stressed the highly tunable character of the eutectic point of these mixtures in the dependence of carboxylic acids’ chain lengths. The factors influencing the phase equilibrium in deep eutectic solvents also were presented in the work by Crema et al. [24]. The authors described a number of HDES using 1,10-phenanthroline as the HBA. The influence of the introduction of an acceptor chlorine atom into thymol, as well as the influence of the steric hindrance of the hydroxyl group, is shown in the example of 2,6-di-tert-butyl-4-methylphenol; however, in this work, only thymol-based HDES were chosen for further extraction, and there was no comparison of these properties with other HDES. Very revealing results related to extraction are given in work [25]. HDES based on tributyl phosphate and TOPO as the HBA increase their extraction capacity with respect to p-hydroxybenzoic acid when switching from octanoic to decanoic and then to dodecanoic acid. However, the authors did not study this phenomenon in detail. A comparison of the extraction properties is also given by Vargas et al. [26] using the example of the extraction of Pt (IV) and Pd (II) HDES ions with TOPO as HBA. It was shown that TOPO in combination with thymol has a relatively lower extraction efficiency compared to TOPO:Decanoic acid, which, however, increases the separation factor of Pt (IV) and Pd (II). As an explanation for this phenomenon, the authors indicate a greater number of hydrogen bonds in the HDES TOPO:Thymol, which reduces the ability of metal ions to complex with TOPO. Conversely, decanoic acid, due to its propensity to form dimers, binds less to TOPO, which as a result does not hinder the extraction; however, a deeper comparative analysis of the effect is difficult due to the strong differences in the structure of the selected HBDs.

Thus, the purpose of this work is to study the possibilities for controlling the extraction ability of TOPO-based HDES as the extractants depending on the donor component. A number of available phenols with different steric hindrances were chosen as the HBD: phenol (Ph), p-tert-butylphenol (PTBP) and 2-isopropyl-5-methylphenol (thymol, Th). The extraction experiments were aimed at the study of the separation process of the Sm/Co pair of samarium–cobalt magnets. This approach should enable the more accurate selection of the HDES’ composition, with the required extraction properties with respect to metals.

## 2. Results and Discussion

### 2.1. Characterization of HDES

In the resulting phase diagram, all the points are the result of the melting of the studied compositions (Figure S1). In the range of compositions with a mole fraction of PTBP from 0.7 to 0.75, a significant decrease in the phase transition temperature was observed with a minimum value of −84.5 °C for a composition with a mole fraction of PTBP of 0.3. A significant decrease in the temperature relative to the initial TOPO (ΔT = 137.7 °C) and PTBP (ΔT = 183.9 °C) was one of the confirmations of HDES’ formation. The composition further described in this article (χPTBP = 0.5) began to melt at 4.6 °C, which is an important indicator, since this HDES can be used for practical purposes at room temperature. It is noteworthy that the eutectic point at a TOPO mole fraction of about 0.7 was also obtained for the previously studied Th:TOPO and Ph:TOPO diagrams [27,28]. The similarity of all three diagrams is due to the same nature of the formation of hydrogen bonds between the P=O group of TOPO and the OH group of phenols; however, the shift of the eutectic point when comparing all three HDES should be in the direction of increasing the mole fraction of TOPO with an increasing ΔH of the melting of phenol [26]. Since the eutectic points in the Th:TOPO and PTBP:TOPO diagrams were not clear enough, and there was no eutectic point at all in the Ph:TOPO diagram, an accurate analysis was not possible. Obviously, with an increase in the molar fraction of TOPO as the extractant, the extraction ability and capacity of the system would increase; however, having fixed the equimolar ratio of HBD and HBA, it was possible to compare all the studied HDES under the same conditions.

^1^H NMR spectra of the original components and the PTBP:TOPO HDES based on them are shown in Figure S2. As can be seen from this figure, there were no new characteristic peaks in the spectrum, which indicates the absence of a chemical reaction between the HDES components. An important change was the disappearance of the signal of the OH group of PTBP in the HDES’ spectrum (5.12 ppm) due to its involvement in the formation of a hydrogen bond. A similar phenomenon also arose when using thymol as the HBD: the signal of the OH group at 4.60 ppm disappeared (Figure S3). In the case of phenol, the signal of the OH group was initially absent from the spectrum (Figure S4), however, as in the other cases, significant shifts of aromatic proton signals corresponding to the hydrogen bond donors were noticeable. This may be due both to the involvement of the phenolic group in the hydrogen bond, and presumably due to secondary interactions between the octyl group of the TOPO and the phenolic rings.

An analysis of the FT-IR spectra (Figures S5–S7) shows the consistency of the composition of the mixture of the two initial components of the HDES. A characteristic signal was the peak at 1143.79 cm^−1^ in the IR spectrum of the pure TOPO, which corresponded to the P=O stretching vibration of the double bond. The shift of this signal to the region of 1138 cm^−1^ in the IR spectra of the PTBP:TOPO, Ph:TOPO and Th:TOPO HDES indicates the involvement of the phosphine oxide oxygen atom in the intermolecular hydrogen bond with the PTBP. Also, on the spectra, there were multiple minor shifts of the peaks corresponding to the stretching vibrations of the C-O, C-H and C=C bonds. This is due to the change in the state of aggregation of the original HDES’ components from a solid to a liquid.

Hydrophobicity is an important property of HDES, since they need to be used as extractants. The fewer HDES’ components that pass into the aqueous phase, the better the selected composition of the system is retained and the fewer organic components from the HDES pass into the spent aqueous solutions as impurities. To study the property described above, samples of three HDES were stirred for 24 h with distilled water in a 1:1 volume ratio. The phases were then separated and analyzed using ^1^H NMR to detect impurities in the respective phases (Figures S8–S10). In the spectrum of the aqueous phase after shaking with HDES Ph:TOPO, the signals of aromatic protons of the phenol ring were noticeable, which was associated with its very high solubility; however, the transition of this component into the phase was insignificant, which was studied in detail in [27]. The solubility of thymol and PTBP is two orders of magnitude lower than that of phenol (0.6, 0.9, and 83 g/L, respectively); therefore, there were no noticeable signals of these components in the NMR spectra of the aqueous phase after shaking with the HDES of Th:TOPO and PTBP:TOPO.

**Figure 1:**
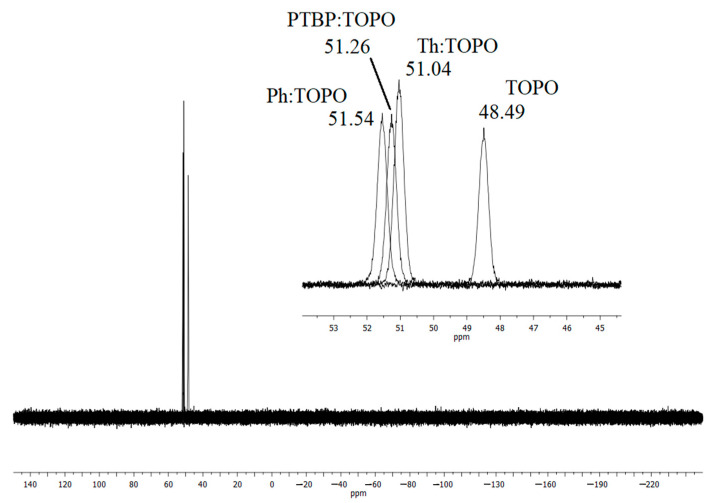
^31^P NMR spectra of hydrophobic deep eutectic solvents based on TOPO.

The most significant changes were seen in the ^31^P NMR spectra (Figure 1) for the HDES. The value of the chemical shift for the phosphorus atom in each HDES (compared with pure trioctylphosphine oxide) indicates the relative difference in the electron density on the phosphorus atoms. Since the redistribution of electron density occurred during the formation of HDES due to the formation of a hydrogen bond, the corresponding peak of the phosphorus atom was shifted to a weak field, while the shift value was proportional to the binding value. Thus, there was a noticeable decrease in the degree of interaction between the oxygen atom of the P=O group and the hydrogen atom of the phenolic group in the series Ph:TOPO > PTBP:TOPO > Th:TOPO.

**Figure 2:**
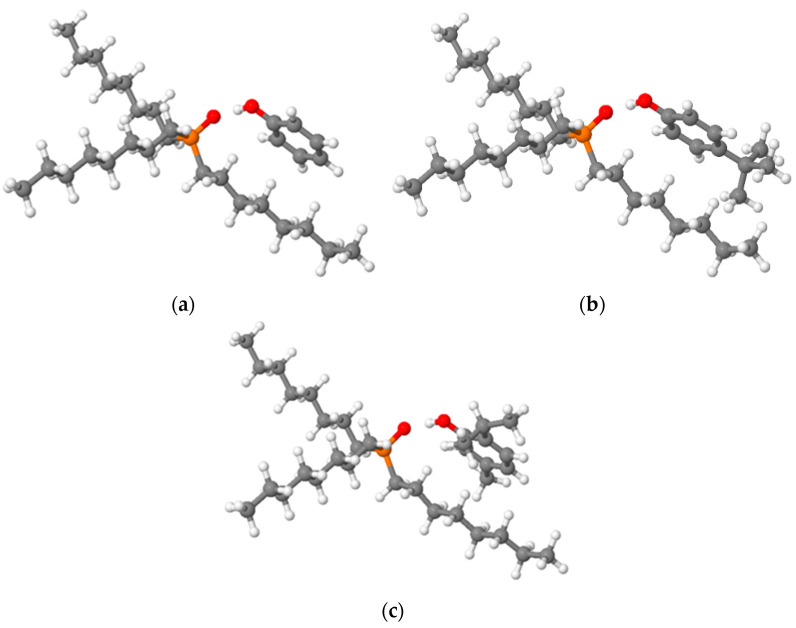
Relaxed geometries of (**a**) phenol-TOPO; (**b**) p-tert-butylphenol-TOPO; (**c**) thymol-TOPO. Red is oxygen, orange is phosphorus, gray is carbon, and white is hydrogen.

For a deeper study of the observed effect of the donor component’s influence on the behavior of the mixture during recovery, DFT calculations at the PBE-D3(BJ)/def2-TZVP theory level were carried out [29,30,31,32,33,34,35]. A polarized continuum model (PCM) with a given dielectric constant ε = 2.6 was used to describe the solvation effect more accurately. The description of the calculation study can be found in the Supplementary Materials. Relaxed geometries for the systems under study are presented in Figure 2.

It should come as no surprise that phenols are bound to TOPO by a non-covalent hydrogen bond O-H···O in the length range of 1.632 to 1.641 Å (Table 1) and form a supramolecular complex. As can be seen from the calculation, the length of the hydrogen bond does not correlate with the sequence described above in the electron density on the phosphorus atom; however, the calculated charge on the oxygen atom of the P=O bond sequentially increased in the series Ph:TOPO < PTBP:TOPO < Th:TOPO, which coincided with the data of the ^31^P spectrum. Undoubtedly, in the case of extraction, the strength of the hydrogen bond indirectly affects the formation of the extraction complex, which was shown in work [19], but in addition to the strength of this bond, it is also important to take into account other interactions between the HBD and HBA. In order to further compare the effects in more detail, in addition to the H-bond, other secondary interactions were also present. There were interactions between the octyl group of the TOPO and the phenolic rings, which were enhanced with phenolic “branching”. The BSSE-corrected energy of the complex in the series phenol—p-tert-butylphenol—thymol decreased by approximately 0.9 kcal/mol. As can be seen from these data, in this case, the influence of the hydrogen bond and the redistribution of the electron density of the P=O bond had a smaller effect on the total energy of the complex than the degree of the branching of the phenol.

The information obtained makes it possible to assume a difference in the experimental extraction properties of the HDES due to the different interaction energies of the components.

### 2.2. Physical Properties of HDES

**Figure 3:**
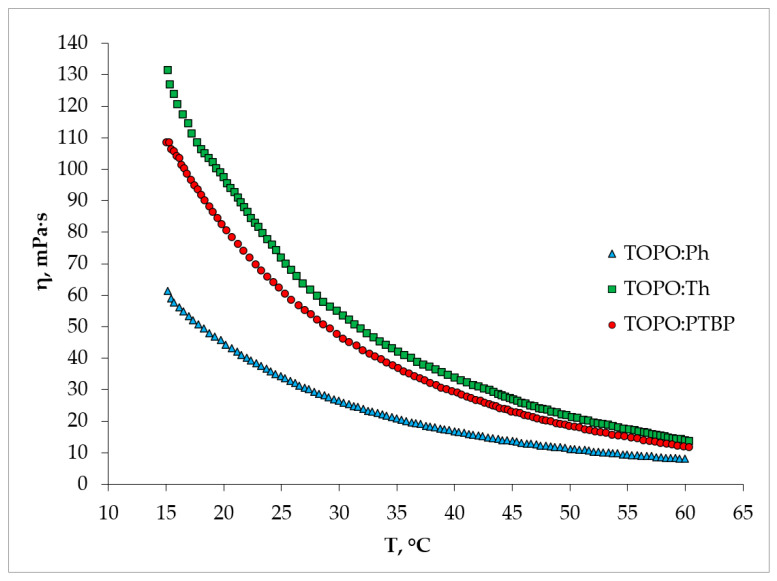
Viscosities of TOPO-based HDES at different temperatures.

The physico-chemical properties of Ph:TOPO and Th:TOPO have been studied previously [27,28]. To determine the feasibility of the PTBP:TOPO HDES in practice, their important physical properties in the temperature range 15 to 60 °C were studied (Figure 3). Solvent viscosity in the liquid extraction process is of particular concern, since it is directly related to the rate of interfacial mass transfer. Studies have shown that, under the standard conditions of the extraction process (25 °C), the viscosity of HDES decreases in the series Th:TOPO > PTBP:TOPO > Ph:TOPO and is 69.93 [28], 62.35 and 42 ± 0.5 mPa·s [27], respectively. As mentioned in the literature [36], the viscosity is highly dependent on the intermolecular interactions between the components of the HDES. The resulting dependence of viscosity on the choice of the hydrogen bond donor is in complete agreement with the data presented above: the lowest complex energy of Th:TOPO correlated with the highest viscosity, and the highest complex energy of the Ph:TOPO density stands for the lowest viscosity.

**Figure 4:**
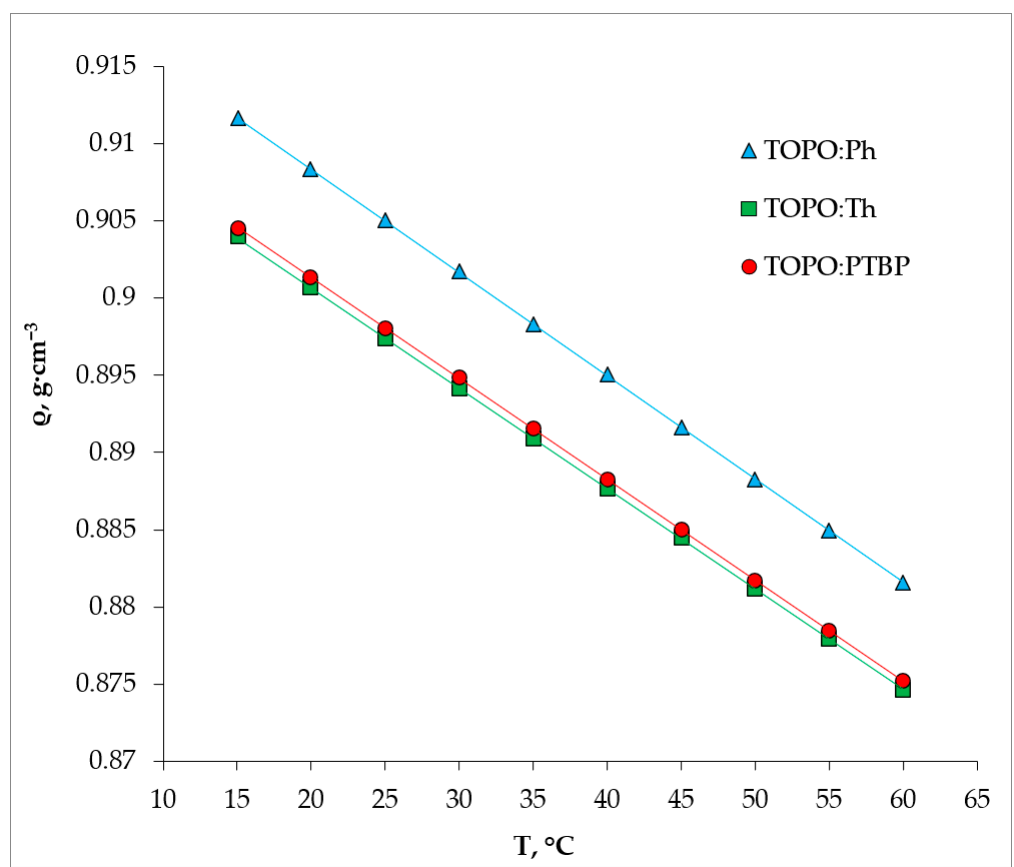
Densities of TOPO-based HDES at different temperatures.

The separation between HDES and the aqueous phase is heavily dependent on the phase density. In the case of nearly-equal densities of HDES and the aqueous phase, a difficult-to-separate emulsion may form. The density of the PTBP:TOPO HDES was measured as a function of the temperature; the experimental data are reported in Figure 4. As expected, the density measured for the HDES linearly decreased with an increasing temperature as a result of thermal expansion at a constant pressure. Using the density data obtained at different temperatures in conjunction with similar data available in the literature [27,28] made it possible to calculate the molar concentration of TOPO in all the HDES, which was 1.8825, 1.6729 and 1.6725 mol/L for the Ph:TOPO, PTBP:TOPO and Th:TOPO, respectively. Density usually depends on the molecular organization and packing [36]. The resulting sequence was probably due to the fact that phenol, being the least sterically hindered, forms a liquid with the smallest free volume. This is also characteristic of the density of pure phenol. The similar structure of thymol and PTBP, as well as an equal molecular weight, is the reason for their almost the same density; however, this slight difference in density between the Th:TOPO and Ph:TOPO can be explained by the fact that thymol is a disubstituted phenol while PTBP is a monosubstituted one.

The refractive index is a parameter that characterizes the electron polarizability of a molecule and is important for understanding the intermolecular interactions or the behaviors of molecules in solution. The dependence of the refractive index of a deep eutectic solvent based on TOPO and PTBP on temperature was studied (Table 2). As can be seen from the table, the HDES’ refractive index values decreased linearly with an increasing temperature in the studied range, which correlated with the nature of the density dependence on the temperature; however, the values were quite close and practically did not depend on the choice of the component. This is probably due to their similar structure and, as a consequence, similar polarizability. Given that the concentration of the components in the mixture was also the same, then according to the Lorentz–Lorenz equation, the refractive indices would be almost equal, which was confirmed by the experimental data [37].

### 2.3. Extraction Experiment

**Figure 5:**
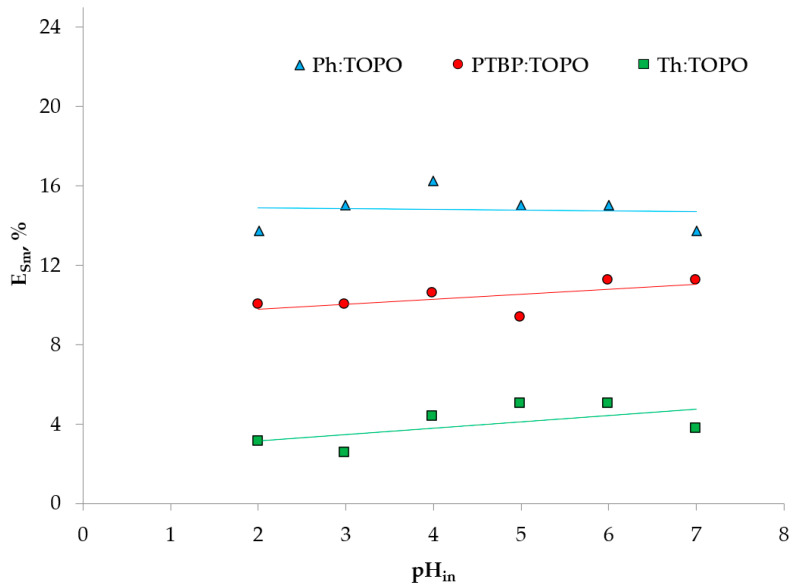
Extraction of Sm (III) ions with HDES based on TOPO at various pH values. The aqueous phase contained 0.01 mol/L Sm (III), O:A = 1:5.

**Figure 6:**
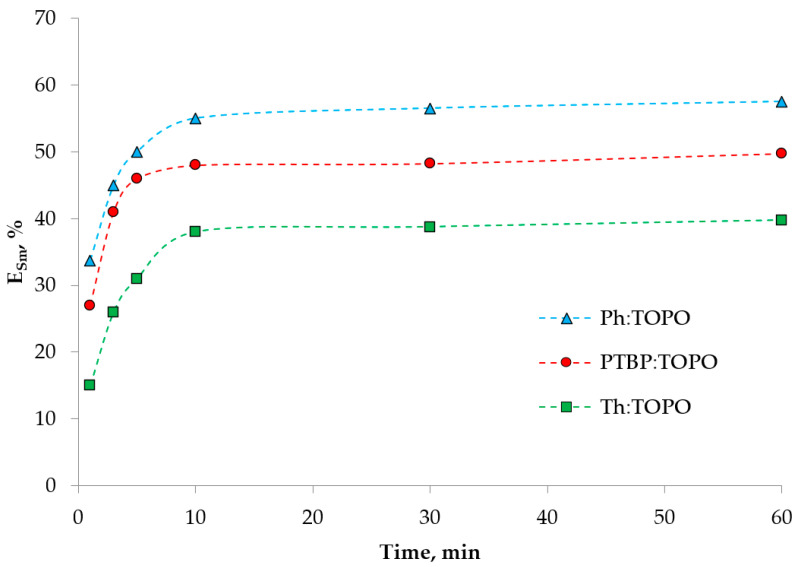
Extraction kinetics of Sm (III) with HDES based on TOPO. Phase ratio O:A = 1:5. The aqueous phase contained 0.1 mol/L Sm (III) and 1 mol/L NaNO3, pH_in_ = 3.

**Figure 7:**
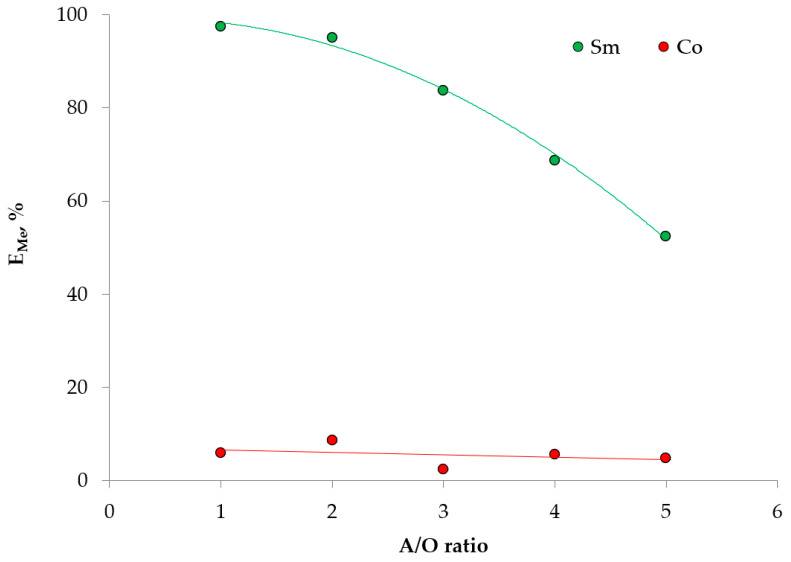
Dependence of the extraction degree of metal ions with Ph:TOPO on the volume ratio of the phases. The aqueous phase contained 0.1 mol/L Me^n+^ and 1 mol/L NaNO_3_, pH_in_ = 3.

A preliminary study of the extraction of ions by the proposed HDES made it possible to determine the conditions for all the subsequent experiments (Figure 5, Figure 6 and Figure 7).

The acidity of the medium did not affect the extraction efficiency of Sm (III) in the extraction systems studied (Figure 5), due to its neutral interfacial distribution mechanism. The neutral mechanism is typical for metal extraction using TOPO. As in previous studies [38,39], the qualitative composition of HDES did not affect the extraction mechanism when using auxiliaries that did not have an extraction ability in relation to metals.

The influence of the phase contact time (in the range from 1 to 60 min) on the extraction efficiency was studied using the example of Sm (III) ions with the selected HDES (Figure 6). The results showed that the establishment of thermodynamic equilibrium in the systems occurred in 10 min. A further increase in the contact time of the phases did not lead to an increase in the extraction degree of Sm (III), suggesting that it is advisable to carry out the extraction for no more than 10 min.

Figure 7 shows the dependences of the extraction degree of Sm (III) and Co (II) ions on variations in the volume ratio of the HDES and aqueous phases, using HDES Ph:TOPO as an example. As can be seen from the graph, the extraction capacity of HDES decreased by a factor of two when the volume of the extractant was reduced by a factor of five, which was a good result and demonstrates the high capacity of the extractant. The ratio O:A = 1:5 was chosen as a model, and all the further experiments were carried out under this condition.

The plots obtained show a difference in the extraction capacity of the HDES used. The relative increase in extraction in the series Th:TOPO < PTBP:TOPO < Ph:TOPO corresponded to approximately 10%, as shown in Figure 6. This effect will be discussed after the further extraction experiments. Figure 7 also shows the high selectivity of this extractant to Sm (III) ions over Co (II). This is consistent with the literature data, where the extraction properties of TOPO in organic solvents are well studied [39].

**Figure 8:**
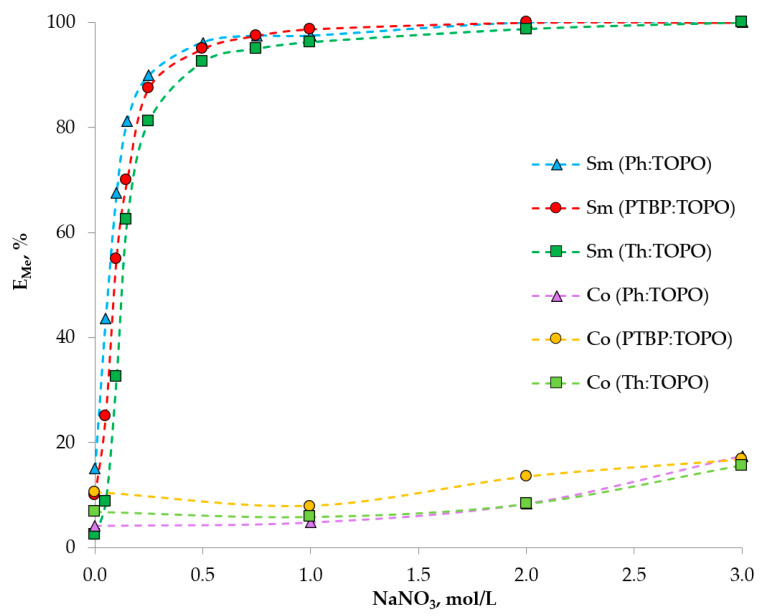
Extraction of Sm (III) and Co (II) ions by TOPO-based HDES at different concentrations of NaNO_3_. The aqueous phase contains 0.01 mol/L Me^n+^, pH_in_ = 3, O:A = 1:5.

**Figure 9:**
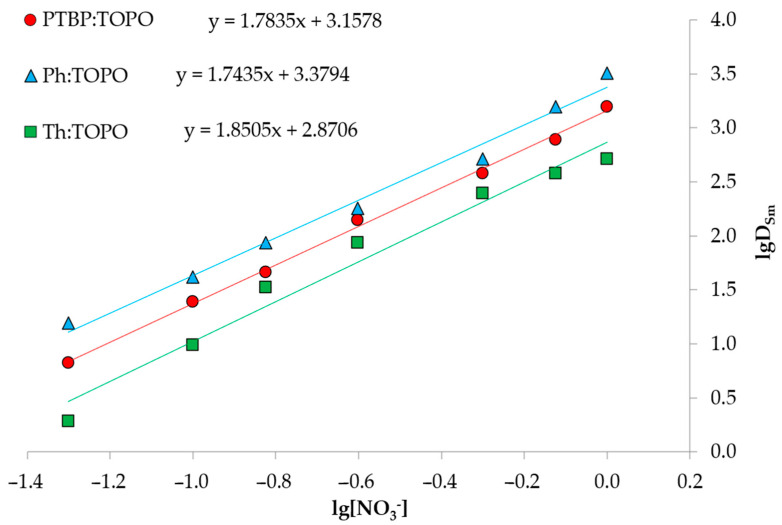
Bilogarithmic dependence of the distribution coefficient of Sm (III) on the NaNO_3_ concentration during HDES’ extraction.

As mentioned above, since TOPO is a neutral extractant, the extraction process will be limited by the formation of coordinated metal complexes with a nitrate ion [39]. A series of further experiments showed the dependence of the recovery rates of Sm (III) and Co (II) ions on the concentration of the nitrate ion in the range from 0 to 3 mol/L (Figure 8). The main parameter influencing the degree of metal extraction was the concentration of NaNO_3_ as a nitrate ion source. For the Co (II), the recovery rate was rather low and did not reach values above 20%. For the Sm (III), a sharp increase in the degree of extraction from 2.5 to 15%, to 97 to 98%, in the given range of NaNO_3_ concentrations was observed. Under the conditions of an initial metal concentration of 0.01 mol/L, pH_in_ = 3 and 1 mol/L NaNO_3_, the Sm/Co partition coefficient reached values > 30 for all the studied HDES. This characteristic of the curve indicates the dependence of the extraction mechanism on the concentration of NO_3_^−^ ions. To analyze, the bilogarithmic dependence of the partition coefficient on the concentration of NO_3_^−^ ions was plotted (Figure 9). The tangent of the slope of the line is approximately two, indicating the involvement of two nitrate ions in the extraction process.

The extraction of lanthanides with trioctylphosphine oxide in nitrate systems occurs through an addition reaction at a metal:extractant ratio of 1:3 [38,40,41]. Since TOPO is a neutral extractant and extracts uncharged metal forms into the HDES phase, the extraction of Sm (III) ions that is considered to be SmOH_2_^+^ [39,42] can be represented in general terms by the following equation:

SmOH_2_^+^_(aq)_ + 2NO_3_^−^_(aq)_ + 3TOPO:HBD_(HDES)_ ↔ SmOH(NO_3_)_2_∙3TOPO:HBD_(HDES)_ (1)

From the above equation, the equilibrium constant can be expressed as:

(2) $$K_{\mathrm{eq}}=\frac{[\mathrm{SmOH}({\mathrm{NO}}_{3}{)}_{2}\cdot 3\mathrm{TOPO}:\mathrm{HBD}{]}_{\left(\mathrm{HDES}\right)}}{[{{\mathrm{SmOH}}_{2}}^{+}{]}_{\left(\mathrm{aq}\right)}\;{[{{\mathrm{NO}}_{3}}^{-}{]}^{2}}_{\left(\mathrm{aq}\right)}\;{{\left[\mathrm{TOPO}:\mathrm{HBD}\right]}^{3}}_{\left(\mathrm{HDES}\right)}}$$

This could be written as:

(3) $$K_{\mathrm{eq}}=\frac{D}{[{{\mathrm{NO}}_{3}}^{-}{{]}^{2}}_{\left(\mathrm{aq}\right)}\;[\mathrm{TOPO}:\mathrm{HBD}{{]}^{3}}_{\left(\mathrm{HDES}\right)}}$$

where:

(4) $$D=\frac{[\mathrm{SmOH}({\mathrm{NO}}_{3}{)}_{2}\cdot 3\mathrm{TOPO}:\mathrm{HBD}{]}_{\left(\mathrm{HDES}\right)}}{[{{\mathrm{SmOH}}_{2}}^{+}{]}_{\left(\mathrm{aq}\right)}}$$

Taking the log of both sides of Equation (6) yields:

lgK_eq_ = lgD − 2lg[NO_3_^−^]_(aq)_ − 3lg[TOPO:HBD]_(HDES)_ (5)

lgD = lgK_eq_ + 2lg[NO_3_^−^]_((aq)_ + 3lg[TOPO:HBD]_(HDES)_ (6)

where HBD stands for Ph, Th or PTBP.

**Figure 10:**
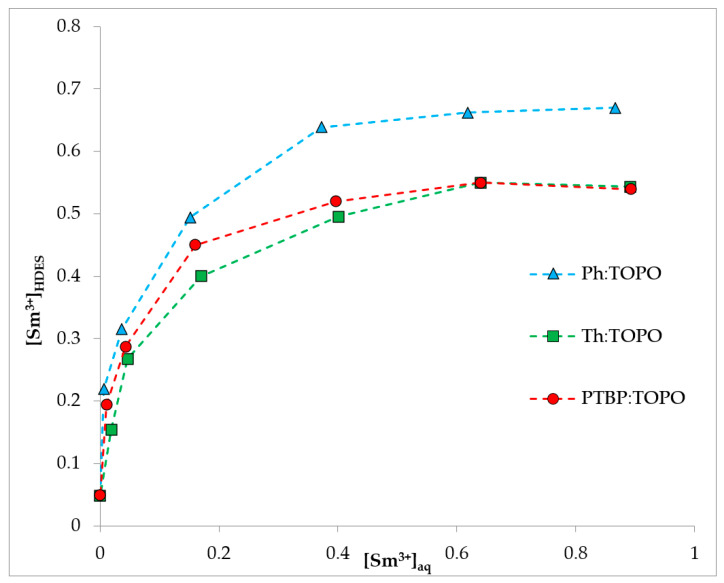
Extraction of Sm (III) ions by TOPO-based HDES as a function of the initial metal concentration. The aqueous phase contained 2 mol/L NaNO_3_, pH_in_ = 3, O:A = 1:5.

To confirm the mechanism of extraction, an isotherm was plotted under the conditions of excess sodium nitrate (Figure 10). As can be seen from the graphs obtained, there were characteristic plateaus; therefore, all the proposed HDES were saturated with metal ions. The concentration of Sm (III) ions in the saturated phase in the case of TOPO:Ph HDES was 0.67 mol/L while the found concentration of TOPO was 1.88 mol/L. The concentration of samarium in the case of the saturated HDES Th:TOPO and PTBP:TOPO phases was approximately 0.55 mol/L, and the found concentration of TOPO in these HDES was 1.67 mol/L; therefore, the molar ratio of Sm (III):TOPO was 1:3 in all cases. In this case, for all the points, the previously described difference in the extraction ability appeared. The simultaneous saturation of HDES based on thymol and p-tert-butylphenol was associated with the same concentration of TOPO in these systems. The phenol-based system had a higher concentration of TOPO, and therefore saturated at a higher metal concentration.

Thus, the graphs clearly show the following relation: the use of a more branched HBD to create HDES based on TOPO reduces the extraction capacity of the system. Based on the results presented in Section 2.1, this phenomenon is not fully characterized by the strength of the non-covalent interaction between the proton of the phenol group and the oxygen of the P=O bond. On the contrary, this trend is consistent with the branching of the hydrogen bond donor used and, as a consequence, with the energy of the complex. At the same time, the steric hindrance of the OH group is also not an important factor affecting the extraction ability of HDES in this case. The availability of the OH group in phenol and PTBP is quite similar; however, the energy of the bound complex and the extraction ability differ quite strongly.

## 3. Materials and Methods

### 3.1. Materials

The specifications of the chemicals used in this work are given in Table S1. All chemicals were used as received from the supplier, without further purification.

### 3.2. Preparation of HDES

All mixtures of TOPO and thymol, and phenol and p-tert-butylphenol were prepared via mixing the HBA (i.e., TOPO) and HBD (i.e., Ph, PTBP and Th) in an equal molar ratio. The mixture of HBD and HBA was then stirred and heated in a polypropylene tube with a stopper using a temperature-controlled shaker, namely, the Enviro-Genie SI-1202 (Scientific Industries, Inc., Bohemia, NY, USA) (with an accuracy of ±0.2 °C), at 60 °C for 30 min until the obtainment of an homogeneous liquid. The prepared HDES were then stored in a glass bottle.

### 3.3. Characterization Methods of HDES

The melting points of the pure components and their mixtures were determined using a differential scanning calorimeter instrument (Mettler Toledo, Greifensee, Switzerland). All the transition temperatures of the HDES were determined in a range of temperatures from −100 to +150 °C, with a heating and cooling rate of 1 °C·min^−1^. The phase transition temperature was determined by the peak onset.

The ^1^H and ^31^P spectra were recorded using a Bruker Fourier 300 HD spectrometer (Bruker, Billerica, MA, USA) using (CD_3_)_2_SO as the solvent. The FT-IR spectra were recorded on an IRTracer-100 spectrometer (Shimadzu, Japan) with a diamond crystal ATR accessory.

The densities of the HDES were determined in the temperature range from 15 to 60 °C using an Anton Paar DMA 1001 (Anton Paar, Gratz, Austria) with a precision of ±0.0001 g·cm^−3^. The refractive indexes of the HDES were measured using an Anton Paar Abbemat 3200 refractometer (Anton Paar, Gratz, Austria) with a precision of ±0.0001. The viscosities of the HDES were measured using a TA HR30 Discovery Series (TA Instruments, New Castle, DE, USA). The relative uncertainty of viscosity was estimated to be 1%.

### 3.4. Metal Extraction

All extraction experiments were carried out in polypropylene tubes with stoppers at T = 25 °C. The volume ratio of the HDES phase to the aqueous phase was 1:5 unless otherwise specified. The tubes with samples of the prepared aqueous phase and HDES were placed in a shaker with temperature control and mixed at 35 rpm until thermodynamic equilibrium. The thermodynamic equilibrium time was determined to be 10 min by varying the mixing time from 1 to 60 min. After mixing and centrifuging (at 2500 rpm for 10 min in the centrifuge CM-6MT (SIA ELMI, Riga, Latvia)), the samples were separated and analyzed. The distribution coefficient (D), extraction degree (E) and separation factor (β) were calculated using Equations (1)–(3):

(7) $$D=\frac{{\left[\mathrm{Me}\right]}_{\mathrm{HDES}}}{{\left[\mathrm{Me}\right]}_{\mathrm{aq}}}$$

(8) $$E\left(\%\right)=\frac{n_{\mathrm{in}}-n_{\mathrm{aq}}}{n_{\mathrm{in}}}$$

(9) $$\beta =\frac{\mathrm{DA}}{\mathrm{DB}}$$

where [Me]_HDES_ and [Me]_aq_ are the equilibrium metal ion concentrations in the HDES and aqueous phases, respectively; n_in_ and n_aq_ are the amounts of metal ions in the initial solution and aqueous solution after extraction, respectively; and D_A_ and D_B_ are the distribution ratios of metals A and B, respectively. All the experimental data were measured three times; the values were averaged with a relative standard deviation of less than 5%.

The concentration of metal ions in the initial solution and in the aqueous phase after separation was detected by ICP-OES (Thermo Scientific ICAP PRO XP, Waltham, MA, USA), and the concentrations of metal ions in the HDES phase were calculated by mass balance. The working solutions of Sm (III) and Co (II) were prepared by diluting the standard solutions.

## 4. Conclusions

Here, HDES PTBP:TOPO was prepared and described for the first time. Along with the previously described HDES Th:TOPO and Ph:TOPO, their extraction abilities with respect to Sm (III) and Co (II) ions were studied. It was shown how components that do not have extraction properties can significantly change the efficiency of the extraction of samarium ions, with the difference in the degree of extraction reaching more than 20% under the same conditions. An increase in the efficiency of the extraction properties in the series Th:TOPO < PTBP:TOPO < Ph:TOPO was revealed. A similar sequence was observed when comparing the physical properties: when using thymol as an HBD, HDES had the highest viscosity and the lowest density. This phenomenon has been explained in terms of the different energies of the HBD:HBA complexes. The interaction of components in the HDES was characterized using computational and spectral methods. The obtained data develop the theory of the fine formation of extraction properties of deep eutectic solvents. The HDES obtained were used as extractants for the efficient separation of Sm (III) and Co (II) ions. Using the extraction of these metals, data on the correlation of the structural features of the HBD with the extraction ability of the corresponding HDES have been shown and systematized.

## Figures and Tables

**Table 1:** Selected calculated properties for phenols–TOPO systems.

HDES	O…H, Å	q_NBO_ (O_TOPO_)	ΔE_complexBSSE_, kcal/mol
Ph:TOPO	1.632	−1.088	−19.73
PTBP:TOPO	1.641	−1.087	−20.60
Th:TOPO	1.639	−1.086	−21.52

**Table 2:** Refractive index (nD) of HDES at different temperatures.

T, °C	Ph:TOPO	PTBP:TOPO	Th:TOPO
15	1.4827	1.4829	1.4832
25	1.479	1.4792	1.4795
35	1.4752	1.4756	1.4758
45	1.4715	1.4718	1.4721
55	1.4679	1.4683	1.4685

## Data Availability

Not applicable.

## Supplementary Materials

The following supporting information can be downloaded at: https://www.mdpi.com/article/10.3390/ijms241814032/s1.
