# Supplementary material for: Sm/Co Magnetic Materials: A Recycling Strategy Using Modifiable Hydrophobic Deep Eutectic Solvents Based on Trioctylphosphine Oxide
Source: Int J Mol Sci. 2023 Sep 13;24(18):14032. doi: 10.3390/ijms241814032 (PMC10531512; doi:10.3390/ijms241814032)
Supplement: Supplementary file 1 [file ijms-24-14032-s001.zip › ijms-2568127-supplementary.pdf]

Supplementary materials

Sm/Co Magnetic Materials: A Recycling Strategy Using Modifiable Hydrophobic Deep Eutectic Solvents Based on Trioctylphosphine Oxide

Nikita A. Milevskii, Inna V. Zinov'eva, Arina V. Kozhevnikova, Yulia A. Zakhodyaeva * and Andrey A. Voshkin

Kurnakov Institute of General and Inorganic Chemistry, Russian Academy of Sciences, Leninsky Prospekt 31, 119991 Moscow, Russia; mna@igic.ras.ru (N.A.M.); iz@igic.ras.ru (I.V.Z.); ak@igic.ras.ru (A.V.K.); voshkin@igic.ras.ru (A.A.V.)

* Correspondence: yz@igic.ras.ru; Tel.: +7-495-9542442

Table S1. Materials

Compound	Supplier	CAS	Purity, wt%*
Trioctylphosphine oxide	Acros	78-50-2	98%
Thymol	Acros	89-83-8	99%
Phenol	Chimmed	108-95-2	99%
p-tert-Butylphenol	Chimmed	98-54-4	99%
HNO_3	Chimmed	7697-37-2	–
$Sm(NO_3)_3\cdot 6H_2O$	Chimmed	13759-83-6	99%
$Co(NO_3)_2\cdot 6H_2O$	Chimmed	10026-22-9	99%
$NaNO_3$	Chimmed	7631-99-4	99%

*Declared by suppliers.

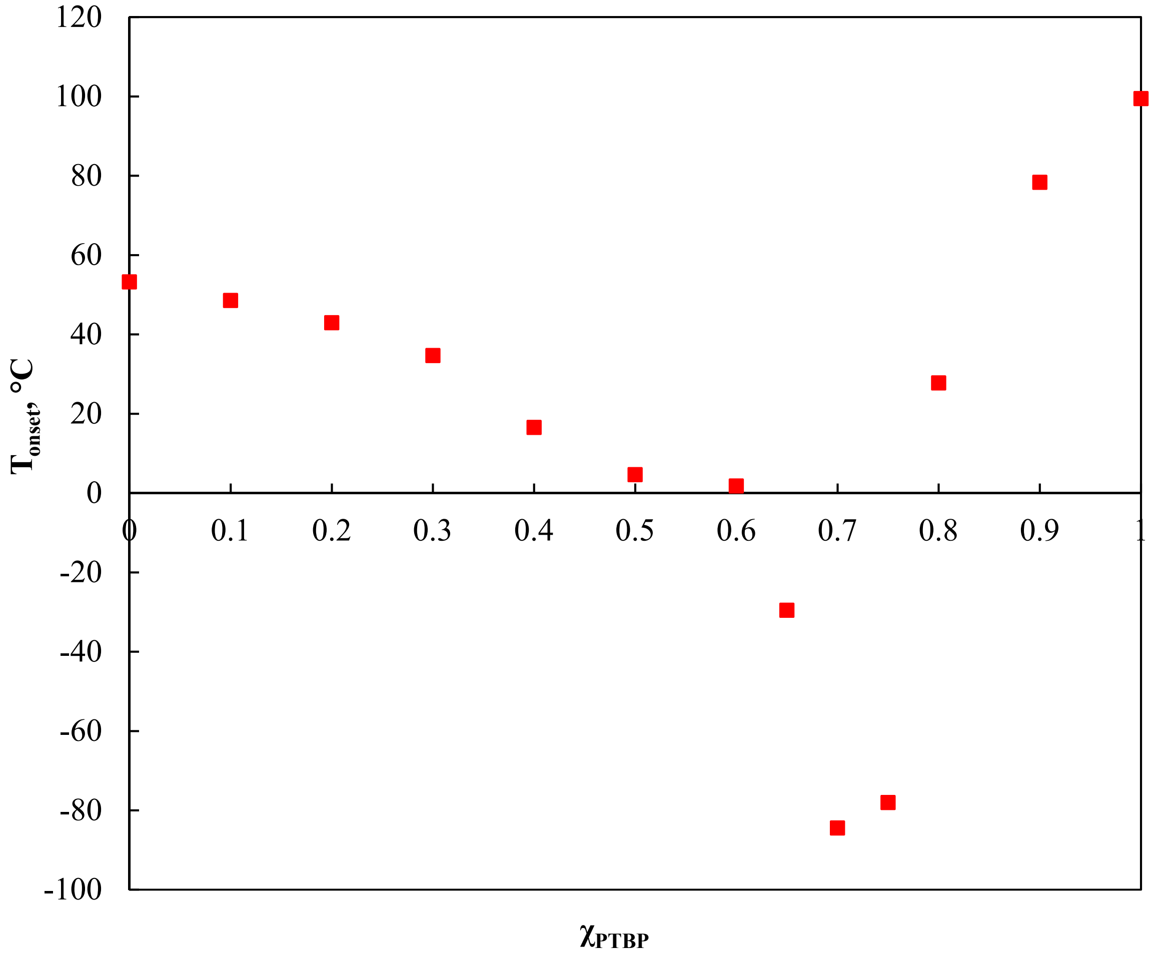

Figure S1. Phase diagram for PTBP:TOPO mixture, constructed from DSC results.

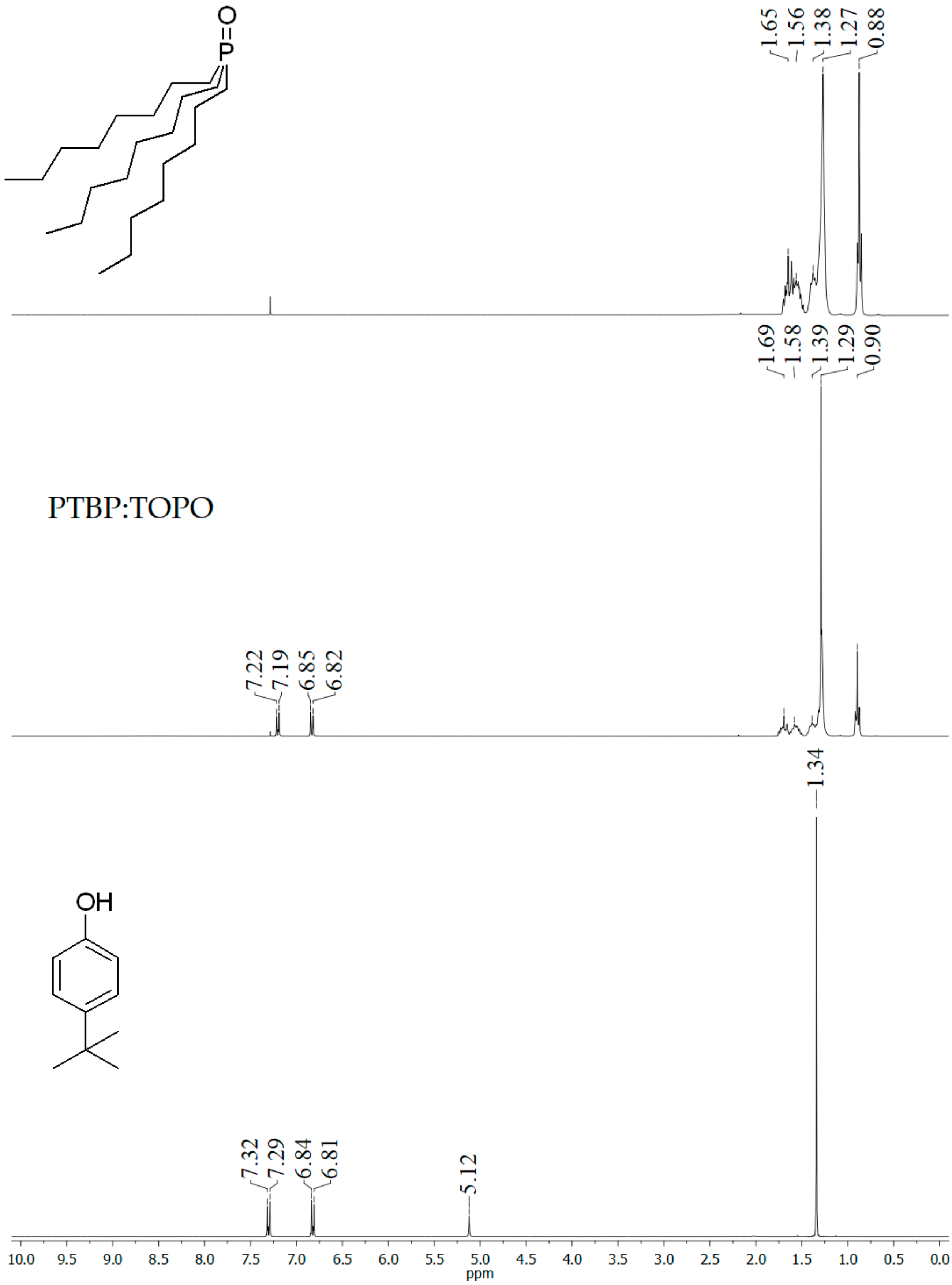

Figure S2. Proton NMR of PTBP:TOPO HDES.

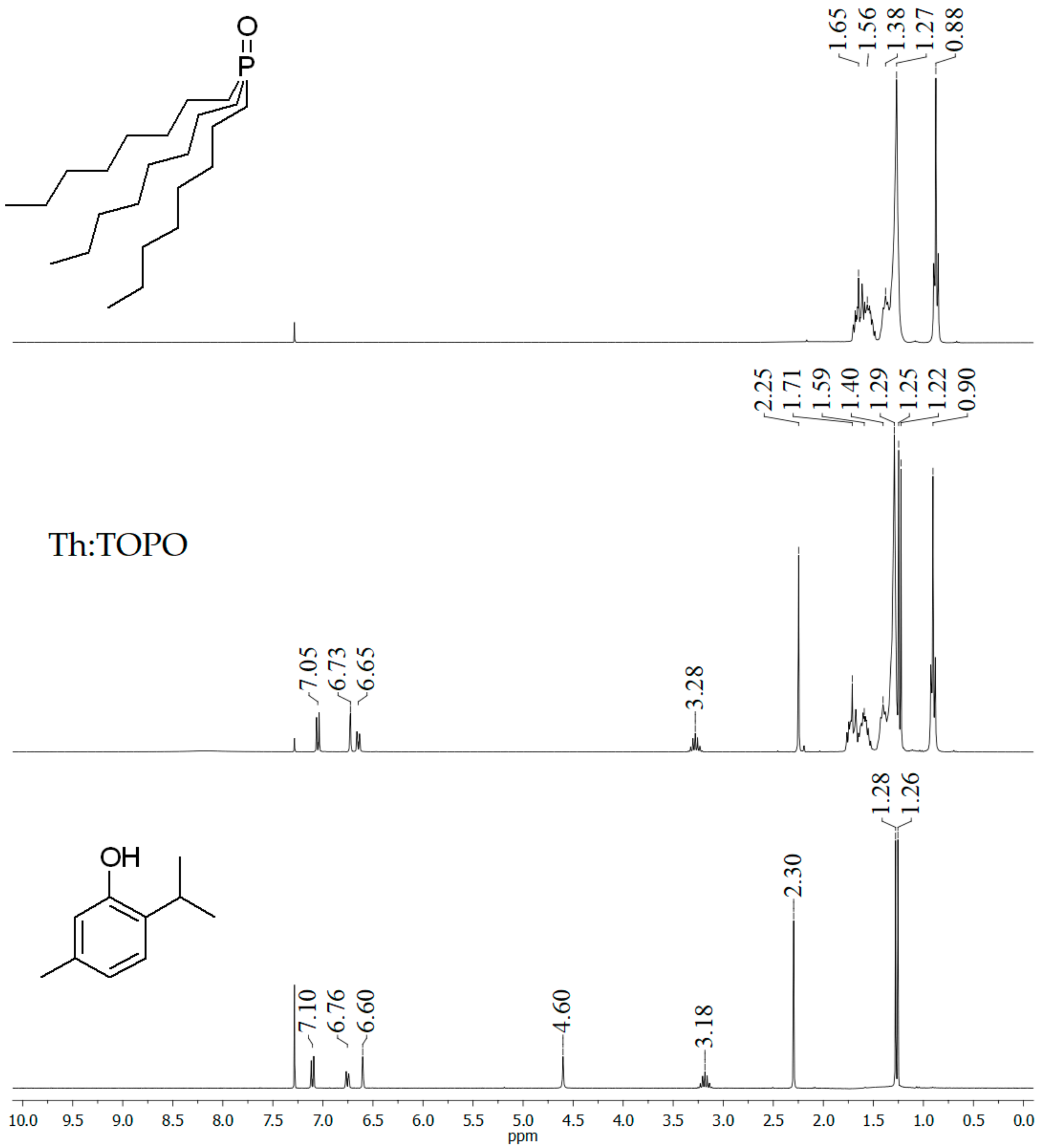

Figure S3. Proton NMR of Th:TOPO HDES.

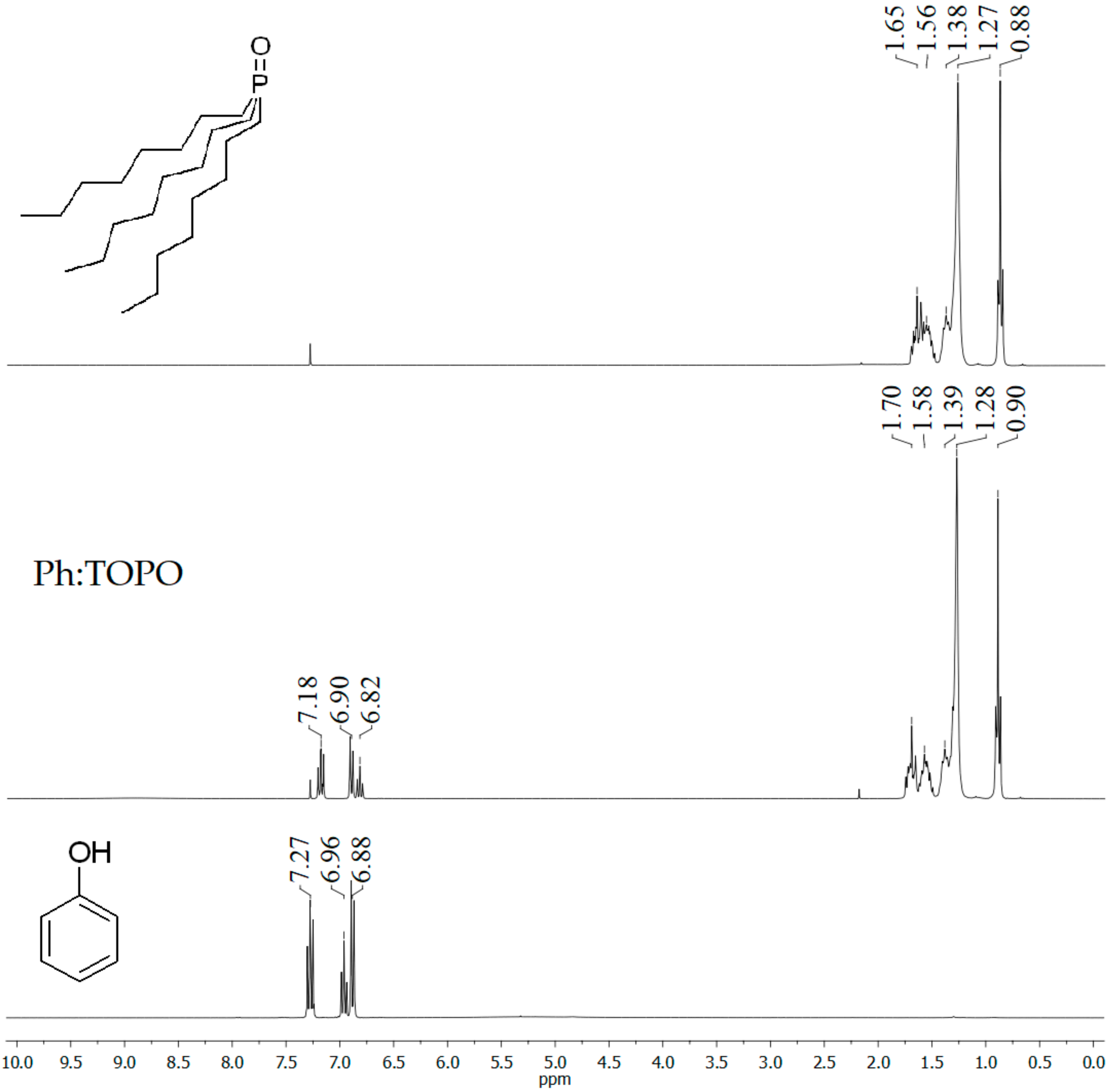

Figure S4. Proton NMR of Ph:TOPO HDES.

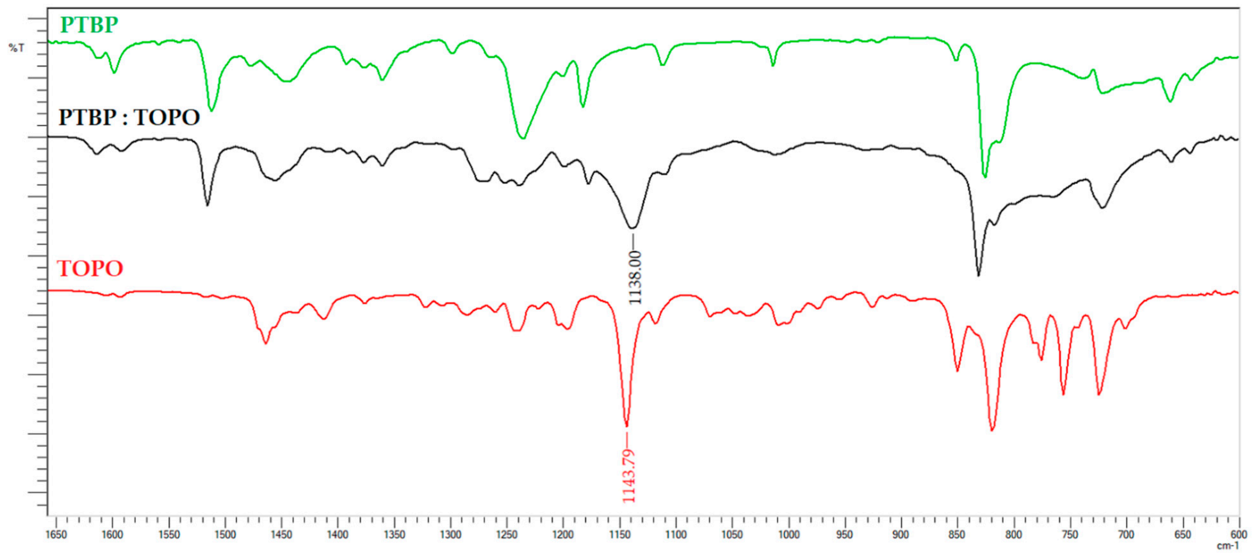

Figure S5. FTIR spectra of PTBP:TOPO HDES.

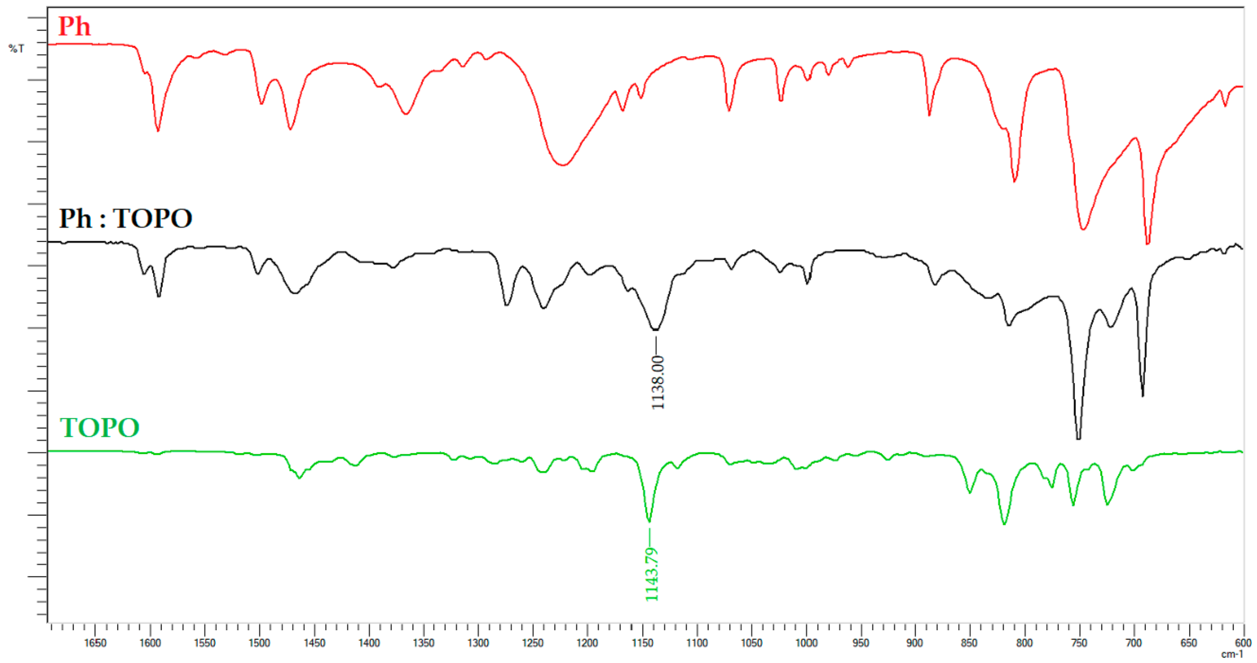

Figure S6. FTIR spectra of Ph:TOPO HDES.

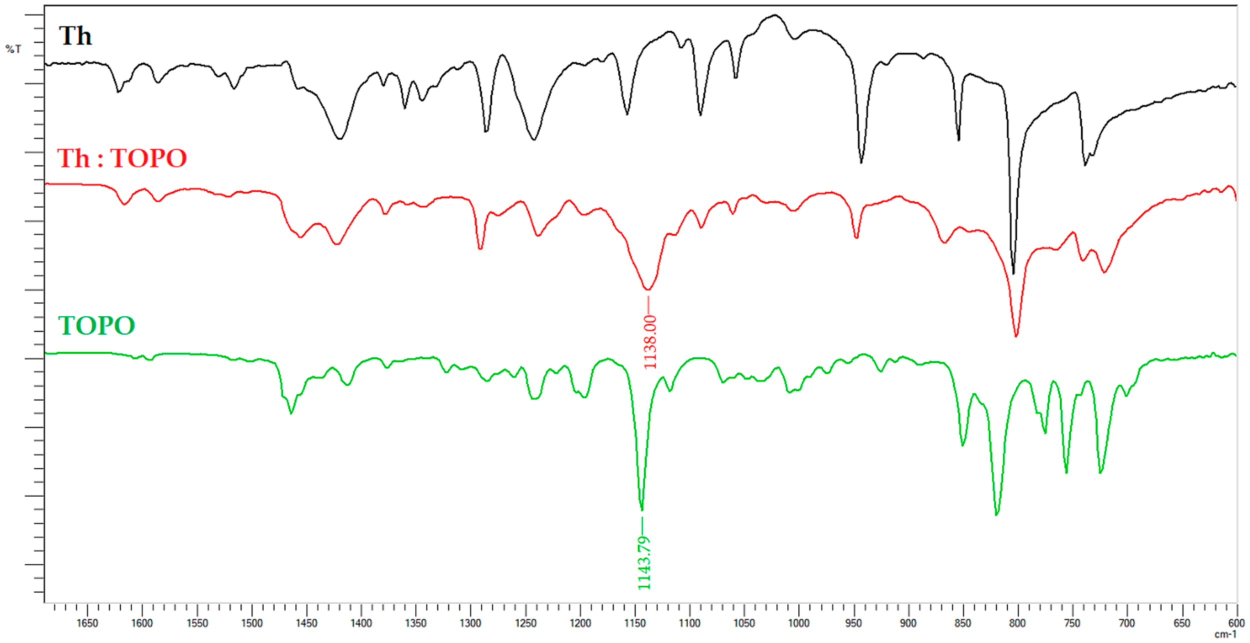

Figure S7. FTIR spectra of Th:TOPO HDES.

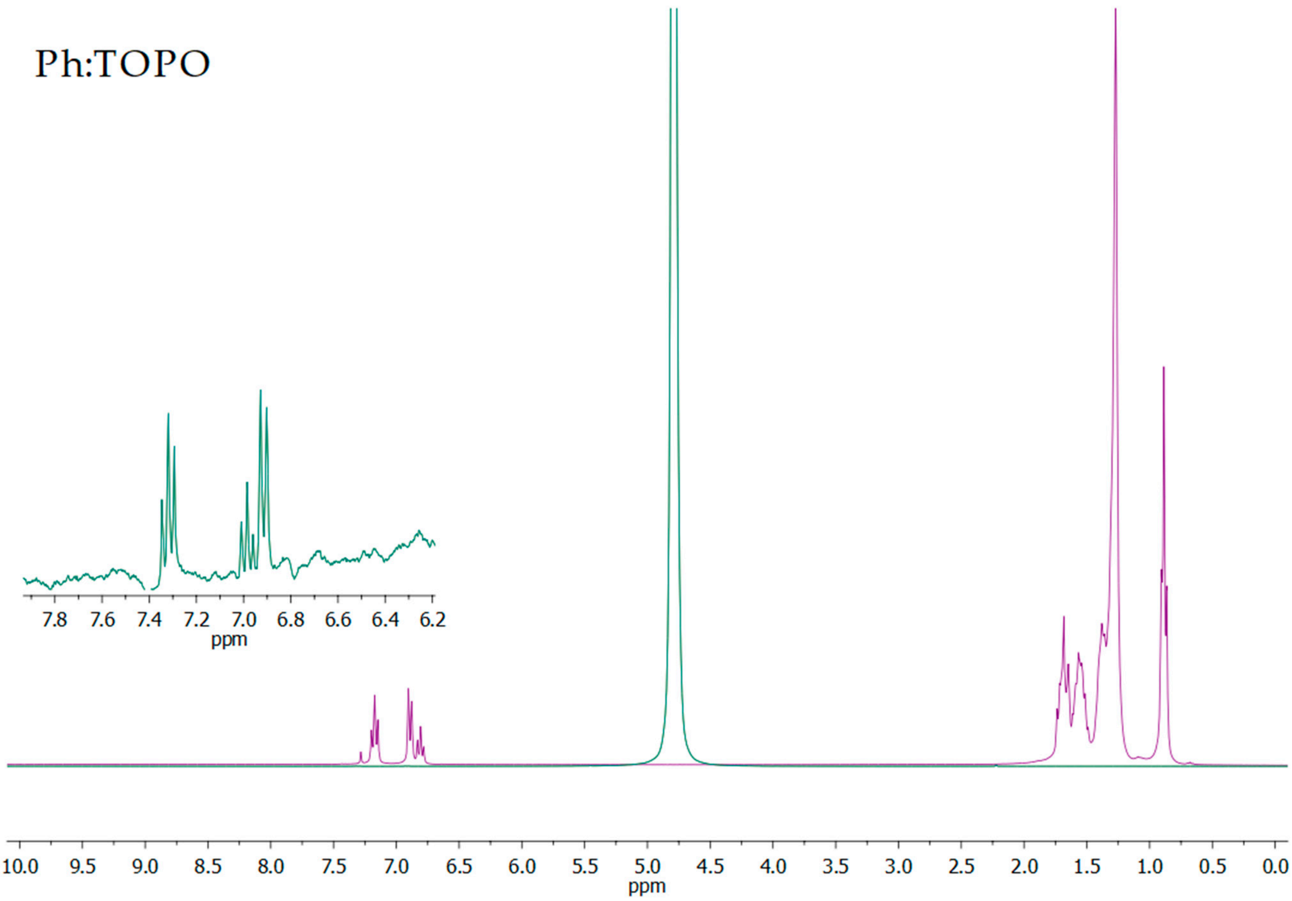

Figure S8. ^{1}H NMR spectra of the aqueous (green line) and HDES Ph:TOPO phases (purple line) after stirring.

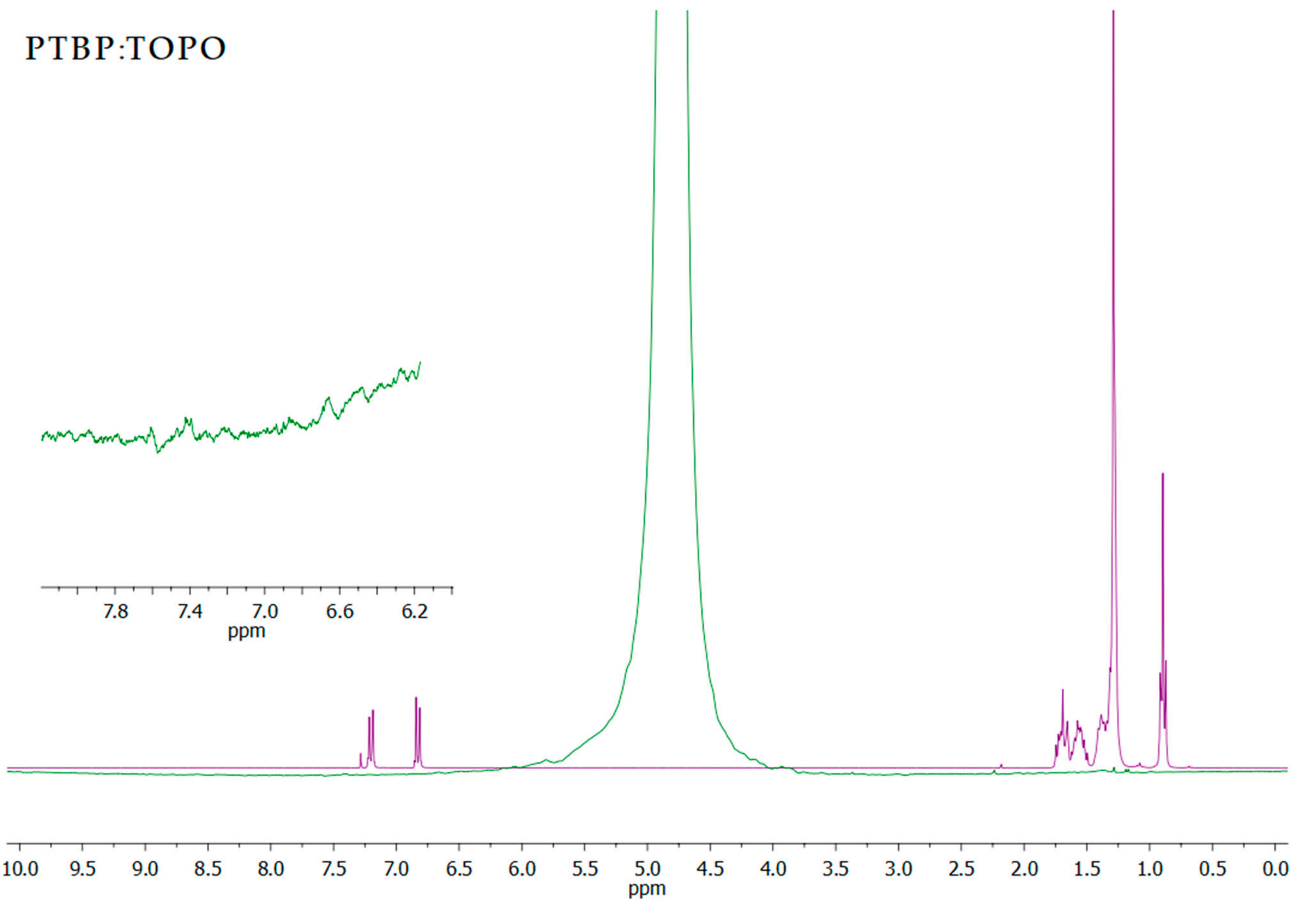

Figure S9. ^{1}H NMR spectra of the aqueous (green line) and HDES PTBP:TOPO phases (purple line) after stirring.

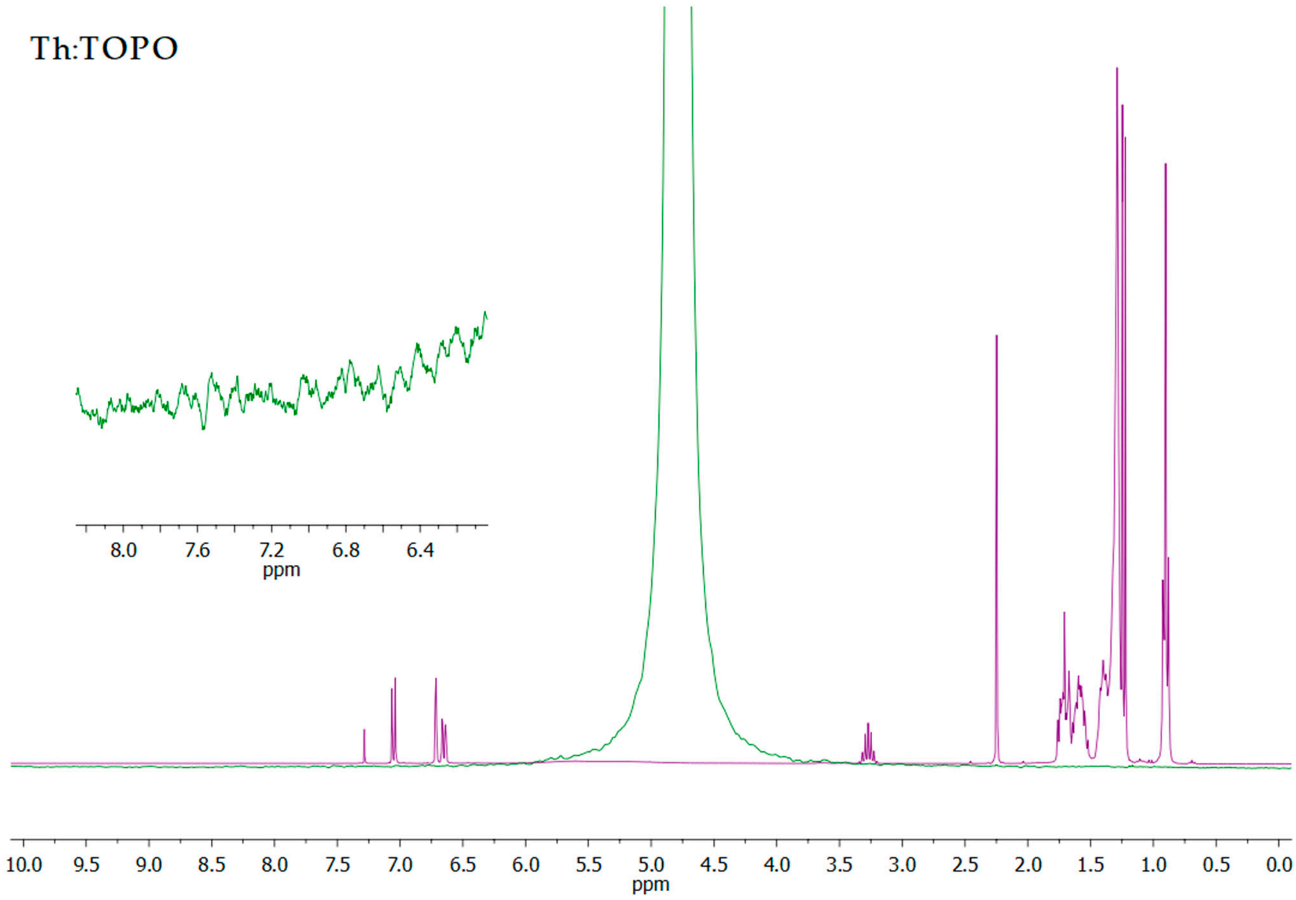

Figure S10. ^{1}H NMR spectra of the aqueous (green line) and HDES Th:TOPO phases (purple line) after stirring.

Computational study

Theoretical calculations: optimization of all molecular geometries, NBO (natural bond orbital) charges, supramolecular complexes energies were performed using Gaussian16 [1] software package at PBE-D3(BJ)/def2-TZVP level of theory. PBE [2] functional and Weigend def2-TZVP [3] basis set has recommended itself as a highly accurate method for modelling small organic molecules [4, 5]. Grimme's D3 dispersion correction with Becke–Johnson damping has been used to better estimation of non-covalent interactions [6]. BSSE correction energies computed using the counterpoise scheme [7]. Tight optimization criteria and ultrafine grids were used in each calculation.

References

[1] Frisch, M.; Trucks, G.; Schlegel, H.; Scuseria, G.; Robb, M.; Cheeseman, J.; Scalmani, G.; Barone, V.; Petersson, G.; Nakatsuji, H. et al.; Gaussian 09, Revision C.01; Gaussian, Inc.: Wallingford CT, 2016.

[2] J. P. Perdew, K. Burke and M. Ernzerhof, Phys. Rev. Lett., 1996, 77, 3865–3868.

[3] F. Weigend and R. Ahlrichs, Phys. Chem. Chem. Phys., 2005, 7, 3297–3305.

[4] M. G. Medvedev, I. S. Bushmarinov, J. Sun, J. P. Perdew and K. A. Lyssenko, Science, 2017, 355, 49–52.

[5] N. Mardirossian and M. Head-Gordon, Molecular Physics, 2017, 115, 2315–2372.

[6] S. Grimme, J. Antony, S. Ehrlich and H. Krieg, J. Chem. Phys., 2010, 132, 154104.

[7] S. F. Boys and F. Bernardi, Molecular Physics, 1970, 19, 553–566.
